# Impact of the built, social, and food environment on long‐term weight loss within a behavioral weight loss intervention

**DOI:** 10.1002/osp4.645

**Published:** 2022-11-03

**Authors:** Selam Tewahade, David Berrigan, Beth Slotman, David G. Stinchcomb, R. Drew Sayer, Victoria A. Catenacci, Danielle M. Ostendorf

**Affiliations:** ^1^ Department of Epidemiology Colorado School of Public Health University of Colorado Anschutz Medical Campus Aurora Colorado USA; ^2^ Division of Cancer Control and Population Sciences National Cancer Institute Bethesda Maryland USA; ^3^ Westat Rockville Maryland USA; ^4^ Department of Nutrition Sciences University of Alabama at Birmingham Birmingham Alabama USA; ^5^ Division of Endocrinology, Metabolism, and Diabetes Department of Medicine University of Colorado Anschutz Medical Campus Aurora Colorado USA; ^6^ Anschutz Health and Wellness Center Department of Medicine University of Colorado Anschutz Medical Campus Aurora Colorado USA

**Keywords:** environmental factors, lifestyle modifications, obesity treatment, socio economic deprivation, weight maintenance

## Abstract

**Background:**

Behavioral weight loss interventions can lead to an average weight loss of 5%–10% of initial body weight, however there is wide individual variability in treatment response. Although built, social, and community food environments can have potential direct and indirect influences on body weight (through their influence on physical activity and energy intake), these environmental factors are rarely considered as predictors of variation in weight loss.

**Objective:**

Evaluate the association between built, social, and community food environments and changes in weight, moderate‐to‐vigorous physical activity (MVPA), and dietary intake among adults who completed an 18‐month behavioral weight loss intervention.

**Methods:**

Participants included 93 adults (mean ± SD; 41.5 ± 8.3 years, 34.4 ± 4.2 kg/m^2^, 82% female, 75% white). Environmental variables included urbanicity, walkability, crime, Neighborhood Deprivation Index (includes 13 social economic status factors), and density of convenience stores, grocery stores, and limited‐service restaurants at the tract level. Linear regressions examined associations between environment and changes in body weight, waist circumference (WC), MVPA (SenseWear device), and dietary intake (3‐day diet records) from baseline to 18 months.

**Results:**

Grocery store density was inversely associated with change in weight (*β* = −0.95; *p* = 0.02; *R* ^2^ = 0.062) and WC (*β* = −1.23; *p* < 0.01; *R* ^2^ = 0.109). Participants living in tracts with lower walkability demonstrated lower baseline MVPA and greater increases in MVPA versus participants with higher walkability (interaction *p* = 0.03). Participants living in tracts with the most deprivation demonstrated greater increases in average daily steps (*β* = 2048.27; *p* = 0.02; *R* ^2^ = 0.039) versus participants with the least deprivation. Limited‐service restaurant density was associated with change in % protein intake (*β* = 0.39; *p* = 0.046; *R* ^2^ = 0.051).

**Conclusion:**

Environmental factors accounted for some of the variability (<11%) in response to a behavioral weight loss intervention. Grocery store density was positively associated with weight loss at 18 months. Additional studies and/or pooled analyses, encompassing greater environmental variation, are required to further evaluate whether environment contributes to weight loss variability.

## Graphical abstract

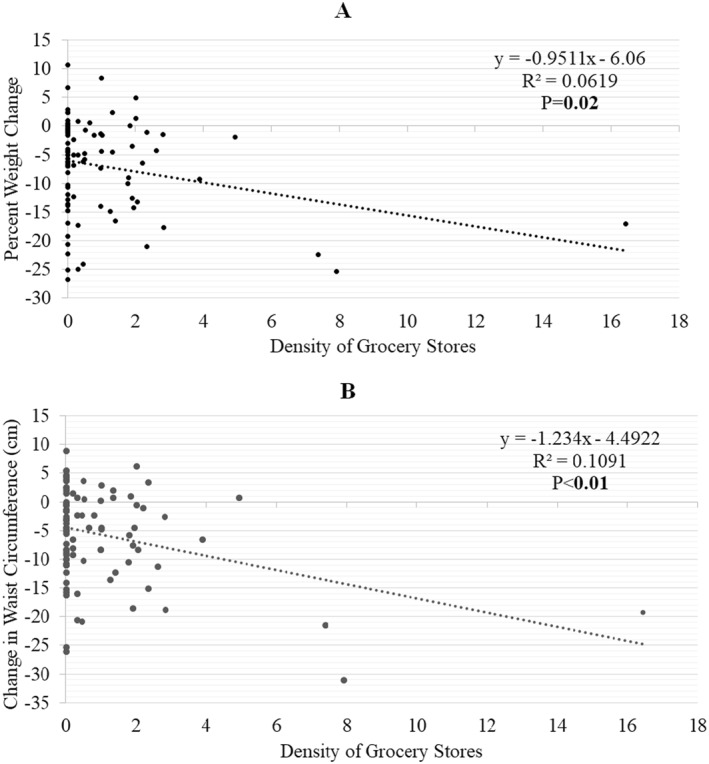

Although built, social, and community food environments can have potential direct and indirect influences on body weight (through their influence on energy intake and physical activity), these environmental factors are rarely considered as predictors of variation in weight loss. This study examined the association between built, social, and community food environments and changes in weight, moderate‐to‐vigorous physical activity, and dietary intake among adults who completed an 18‐month behavioral weight loss intervention. Grocery store density was positively associated with weight loss at 18 months and participants living in less walkable neighborhoods had lower levels of moderate‐to‐vigorous physical activity at baseline but greater increases in moderate‐to‐vigorous physical activity at follow‐up.

## 1 INTRODUCTION

Despite the short‐term effectiveness of lifestyle interventions for weight loss, many individuals regain significant weight within a 1‐year period and there is significant inter‐individual variability in weight loss response.^1^ The National Institutes of Health (NIH) Accumulating Data to Optimally Predict obesity Treatment (ADOPT) Core Measures Project was created to identify factors that predict this variability in response to obesity treatment. ADOPT identifies four domains of focus: biological, behavioral, psychosocial, and environmental.^2^ While several studies have evaluated the contribution of biological, behavioral, and psychosocial factors, fewer have evaluated the role of environment.^3^

The built environment describes human‐made aspects of community design and includes urbanicity (rural vs. urban) and walkability (how friendly an area is for walking).^4^ The social environment describes the makeup of a neighborhood's culture, groups, relationships, and social processes and includes factors like crime (crimes against persons and property) and Neighborhood Deprivation Index (NDI; composed of 13 social economic status (SES) factors).^5^ Lastly, the community food environment is a broad term that describes the distribution, number, type, and location of food sources and includes density of convenience stores, grocery stores, and limited‐service restaurants (LSRs).^6^ It has been hypothesized that environment can influence obesity outcomes.^7^ Observational studies have found that obesity was inversely associated with walkability,^8^ urbanicity (with more urban populations having a lower prevalence of obesity),^9^ SES,^10^ and the presence of grocery stores,^11^ but positively associated with increasing crime,^12^ and presence of convenience stores.^11^ However, the role of environment on weight loss outcomes within the context of a behavioral weight loss intervention has not been well established.

There have been three prior interventional studies that have evaluated the role of environment. Mench et al. found that rural versus urban status did not moderate weight loss or changes in self‐reported physical activity (PA) among a sample of 492 adults who participated in a 6‐month weight loss intervention.^13^ In another study of 114,256 participants from the U.S. Department of Veterans Affairs (VA) MOVE! weight management program, there was no association between walkability, park access, or fitness facility access and weight change at 6, 12, 18, or 24 months.^14^ In terms of the social environment, a third study by Mendez et al. found no association between SES (poverty rate, neighborhood income) and changes in weight over 6 months during a behavioral weight loss intervention among 127 adults.^15^ Lastly, while the community food environment moderated weight loss response among men, but not women, in the VA MOVE! program at 6 months,^16^ community food environment was not associated with changes in weight at 24 months.^17^ One explanation for this lack of association between environment and weight loss outcomes is that factors other than environment, such as psychosocial (motivation, self‐regulation), biological (changes in resting energy expenditure, appetite) and/or behavioral (sleep, timing of eating) may have a greater contribution to weight loss outcomes.^2^ Alternatively, the lack of association could be due to the heterogeneity in methods^18^ and/or a lack of focus on local environmental supports within the behavioral intervention content. Thus, additional research is needed to explore the association between environment and responses to behavioral weight loss interventions.

The purpose of this study was to conduct a secondary analysis of data from an 18‐month behavioral weight loss intervention to explore whether environmental factors, recommended by ADOPT,^3^ are associated with changes in weight at 18 months. The hypotheses were that 1) greater levels of urbanicity and/or walkability would be associated with greater weight loss, 2) higher levels of crime and/or NDI would be associated with less weight loss, and 3) density of convenience stores, and/or LSRs would be associated with less weight loss, while density of grocery stores would be associated with greater weight loss at 18 months. The association between environment and changes in waist circumference (WC), PA, and dietary intake were also explored.

## 2 METHODS

### 2.1 Description of the behavioral weight loss trial

A full description of the weight loss trial (NCT01985568) and primary results were published previously.^19^ All participants provided written informed consent. Briefly, 170 adults with overweight/obesity (age 18–55 years, Body Mass Index (BMI) 27–42 kg/m^2^, 84% female) were randomized 1:1, stratified by sex, to receive one of two 18‐month group‐based behavioral weight loss interventions: standard behavioral therapy (Standard) or sequential behavioral therapy (Sequential). Both randomized groups received an identical 18‐month group‐based behavioral weight loss program (weekly group meetings during months 0–6 followed by monthly group meetings during months 7‐18 led by a registered dietitian), including a reduced calorie diet (1200–1800 kcal/day).^19^ Targeted macronutrient content was 20%–30% fat, 50%–55% carbohydrates, and 20%–25% protein. Randomized groups differed only in the timing of exercise initiation. The Standard group received a supervised exercise program, progressing to 300 min/week of moderate intensity erobic activity during months 0–6, followed by unsupervized exercise during months 7–18. The Sequential group was asked to refrain from changing their exercise habits during months 0–6 and received an identical supervised exercise program during months 7–12, followed by unsupervized exercise during months 13–18. On completion of the 6‐month supervised exercise phase, participants in both groups were instructed to continue 300 min/week of moderate intensity activity and were provided continued access to the exercise facility for the remainder of the study. At 18 months, there were no differences between the Standard and Sequential in changes in weight, moderate‐to‐vigorous PA (MVPA), or energy intake (EI).^19^

### 2.2 Analytical sample

Participants were included if they completed the 18‐month intervention (n = 120, 71% completion rate). Participants were excluded if they changed their home address during the intervention (n = 27). Thus, of 170 participants randomized, 93 were included in the analysis.

### 2.3 Anthropometrics

The primary outcome was change in weight (%) from baseline to 18 months. Body weight was measured using a calibrated digital scale (to the nearest 0.1 kg). Height was measured with stadiometer. WC (cm) was measured at the level of the superior iliac crest.

### 2.4 PA and sedentary behavior

PA was measured over 7 consecutive days using the SenseWear Mini Armband (version 7.0; BodyMedia Inc., Pittsburgh, Pennsylvania). To be included in PA analyses, participants must have had ≥4 valid days, including ≥1 valid weekend day at baseline and 18 months (n = 80). A day was considered valid if the participant wore the device for ≥22.8 h/day, as published previously.^19^ Average daily steps and average daily time (minutes/day) spent sedentary (<1.5 METs), in light‐intensity PA (1.5–3.0 METs), and in MVPA (≥3 METs) were quantified using the manufacturer's proprietary algorithm.

### 2.5 Dietary energy and macronutrient intake

Dietary EI (kcal/d) and macronutrient intake (% kcal from fat, carbohydrates, and protein) were assessed using 3‐day diet records. Diet records were analyzed using Nutrition Data System for Research (version 2016; Nutrition Coordinating Center, University of Minnesota, Minneapolis, Minnesota) by blinded core laboratory staff.

### 2.6 Environment variables

Each participant self‐reported their home address at baseline and any changes in home address at the 18‐month visit. Home address was used to determine participant geolocation (Census geocoding services).^20^ A TractID code was developed by concatenating the state Federal Information Processing Standard (FIPS) code, county FIPS code, and TractID to create an 11‐digit TractID. Participant's TractID was then merged with environmental datasets for each tract in Colorado. For additional methodological details for each environment variable listed below, readers are referred to Saelens et al.,^3^ Slotman et al.,^21^ and the ADOPT Core Measures Environment Domain website.^22^ All environmental variables were analyzed at the census tract level.

### 2.7 Built environment

#### 2.7.1 Urbanicity

Urbanicity is a six category variable^21^ using National Center for Education Statistics urban/rural locale definitions, applied to Census urban/rural population data.^23^ Due to low participant counts in mixed (n = 5) and rural (n = 2) urbanicity, those categories were removed from analyses and treated as missing.

#### 2.7.2 Walkability

Walkability is composed of measures from block‐level variables based on the 2010 census (2013 National Walkability Index dataset).^21^ Block‐level data came from the Environmental Protection Agency's Smart Location Mapping project^24^ and was aggregated into tract‐level variables. Walkability was examined both as a continuous variable and a categorical variable, categorized by tertiles based on the analytical sample (low: n = 26, 9.6 ± 2.8 walkability score; medium: n = 26, 12.9 ± 0.5 walkability score; and high: n = 28, 15.1 ± 0.8 walkability score).

### 2.8 Social environment

#### 2.8.1 Crime

Crime data were purchased from Applied Geographic Solutions for Colorado in 2019. This dataset includes county names, population, total crime, personal crime (murder, rape, robbery, assault), and property crime (burglar, larceny, motor vehicle theft) by census tract. Per the licensing agreement with Applied Geographic Solutions, the study team was required to analyze the data using categories. Crime was sorted by low to high and secondarily by personal crime rate as several tracts had the same crime rate.^21^ Cumulative population was then calculated as the tract's population plus the previous tract's cumulative population. The ration of cumulative population to one third of the total analytical sample population was calculated to create the crime tertiles ratio. A tract was defined as “low” crime for crime tertile ratio ≤1.0; “medium” for crime tertile ratios >1.0 and ≤ 2.0; and “high” crime for crime tertile ratios ≤3.0.

#### 2.8.2 Neighborhood Deprivation Index

NDI was created using factor analysis of tract‐level variables at the national level.^21^, ^22^ NDI is composed of 13 SES factors broken down into four different categories: wealth and income, education, occupation, and housing conditions.^22^ All variables used to create NDI were obtained from the Census Bureau's 5‐year American Community Survey data for 2013–2017.^22^ NDI ranges from −2.5 to +1.9, which is further categorized into quintiles, weighted by the census tract population (such that 20% of the population is in each quintile group). Categories include: least deprivation, below average deprivation, average deprivation, above average deprivation, most deprivation, and NDI not specified. NDI was analyzed based on quintiles to improve interpretability of results. Participants in the “NDI not specified” category (n = 3) were treated as missing in analyses.

### 2.9 Community food environment

Counts of convenience stores and grocery stores were obtained from historical commercial business listings for the specified Standard Industrial Classification codes and chain names from the year 2019 for the state of Colorado from Data Axle USA^®^ as outlined by Jones et al.^25^ Historical listings for the same state and time period for LSRs were purchased from Dun & Bradstreet.^25^ Density of each food outlet type was then summarized by each tract for Colorado (number of stores/land area, mi^2^).^21^ In addition, the density of food outlet types for each tract, plus their neighboring tracts, was calculated to account for the idea that participants may shop for food outside of their immediate tract. This variable was subsequently used in sensitivity analyses.

### 2.10 Assessment of covariates

Age, sex, race, ethnicity, and education were self‐reported at baseline. Randomized group was assigned at baseline. Number of check‐ins to the fitness center were tracked for each study participant and calculated as [total number of check‐ins/18 months] in the Standard group, and [total number of check‐ins/12 months] in the Sequential group.

### 2.11 Statistical analysis

Data were analyzed using SAS software (Version 9.4©, SAS Institute Inc., Cary, NC, USA). Baseline demographic and clinical characteristics were summarized using descriptive statistics. Several covariates (age, sex, race, ethnicity, and randomized group) were tested for confounding using the classical definition: if the covariate was significantly associated with both the exposure and the outcome, but not on the causal pathway, then it was included as a confounder in analyses. A completer's analysis was chosen to ensure that all participants included in the analysis did not change their home address during the duration of the 18‐month intervention. Given that the primary exposure of interest was the participant's environment, defined at the census track level, a completer's analysis ensured that environmental exposures were constant throughout the 18‐month intervention. Home address was only self‐reported at baseline and 18‐month; thus, for participants who withdrew prior to the end of the study, it was unknown whether their home address changed during the 18‐month intervention. Simple linear regression was used to assess the association between each environment variable and change in each outcome at 18 months. Lastly, post‐hoc analyses were conducted using linear regression to explore whether 1) baseline levels of MVPA moderated the association between walkability and changes in MVPA over 18 months, and 2) average number of fitness center check‐ins was correlated with walkability. This study was approved by the Colorado Multiple Institutional Review Board.

## 3 RESULTS

### 3.1 Study participant characteristics

Baseline demographic characteristics of the analytical sample (n = 93) were similar to that of the randomized sample (n = 170, Table 1), except for age. Those excluded from the analysis (n = 77) were, on average, younger (mean ± SD; 36.5 ± 9.6 years) compared to the analytical sample (41.5 ± 8.3 years; t value −3.63; *p* < 0.01). Average weight loss at 18 months was 9.1 ± 7.4%.

**TABLE 1:** Baseline characteristics^a^

Baseline characteristics	Subjects randomized (n = 170)		Sample included in analysis (n = 93)	
	n	Count (%)/Mean ± SD	n	Count (%)/Mean ± SD
Randomization	170		93	
Sequential		71 (50.71)		48 (51.61)
Standard		69 (49.29)		45 (48.39)
Age (years)	170	39.3 ± 9.2	93	41.5 ± 8.3
Weight (kg)	170	95.7 ± 15.5	93	94.7 ± 15.4
BMI (kg/m^2^)	170	34.4 ± 4.1	93	34.4 ± 4.2
Waist circumference (cm)	170	107.0 ± 10.7	93	106.6 ± 10.3
Sex	170		93	
Female		142 (83.53)		76 (81.72)
Male		28 (16.47)		17 (18.28)
Race	170			
White		132 (77.65)	93	70 (75.27)
Black		28 (16.47)		16 (17.20)
Other		10 (5.88)		7 (7.53)
Ethnicity	170		93	
Hispanic or latino		42 (24.71)		25 (26.04)
Not hispanic or latino		128 (75.29)		68 (73.12)
Education level	170		93	
HS/GED		14 (8.28)		7 (7.53)
Some college		43 (25.44)		22 (23.66)
College		112 (66.27)		64 (68.82)

^a^ Abbreviations are as follows: BMI: Body Mass Index; HS: High School; GED: General Education Development.

### 3.2 Environmental data

None of the tested covariates (age, sex, race, ethnicity, education, and randomized group) were associated with changes in outcome variables, thus these covariates were not considered further in these analyses. Environmental data are described in Table 2. Within the sample of 93 participants, 72 tracts were represented.

**TABLE 2:** Geographic variable distribution

Environmental variable	Sample included in analysis (n = 93)
	Count (%)/Mean ± SD
Urbanicity	
City	64 (68.82)
Suburb	22 (23.66)
Rural^b^	2 (2.15)
Mixed^b^	5 (5.38)
Walkability score	12.5 ± 2.8
Crime tertile	
High	32 (34.56)
Medium	30 (32.26)
Low	31 (33.33)
NDI quintiles^a^	
Least deprivation	31 (33.33)
Below average deprivation	17 (18.28)
Average deprivation	21 (22.58)
Above average deprivation	10 (10.75)
Most deprivation	11 (11.83)
NDI not at all^a^ ^,^ ^b^	3 (3.23)
Community food environment	
Convenience store density	0.6 ± 0.9
Convenience store density + neighbors	0.4 ± 0.3
Grocery store density	1.0 ± 2.2
Grocery store density + neighbors	0.7 ± 0.8
LSR density^a^	2.8 ± 3.4
LSR density^a^ + neighbors	2.2 ± 1.8

^a^ Abbreviations are as follows: NDI: National Deprivation Index; LSR: Limited Service Restaurants.

^b^ These categories were removed from further analyses due to the small sample sizes.

### 3.3 Association between environment and change in anthropometric outcomes

**FIGURE 1:**
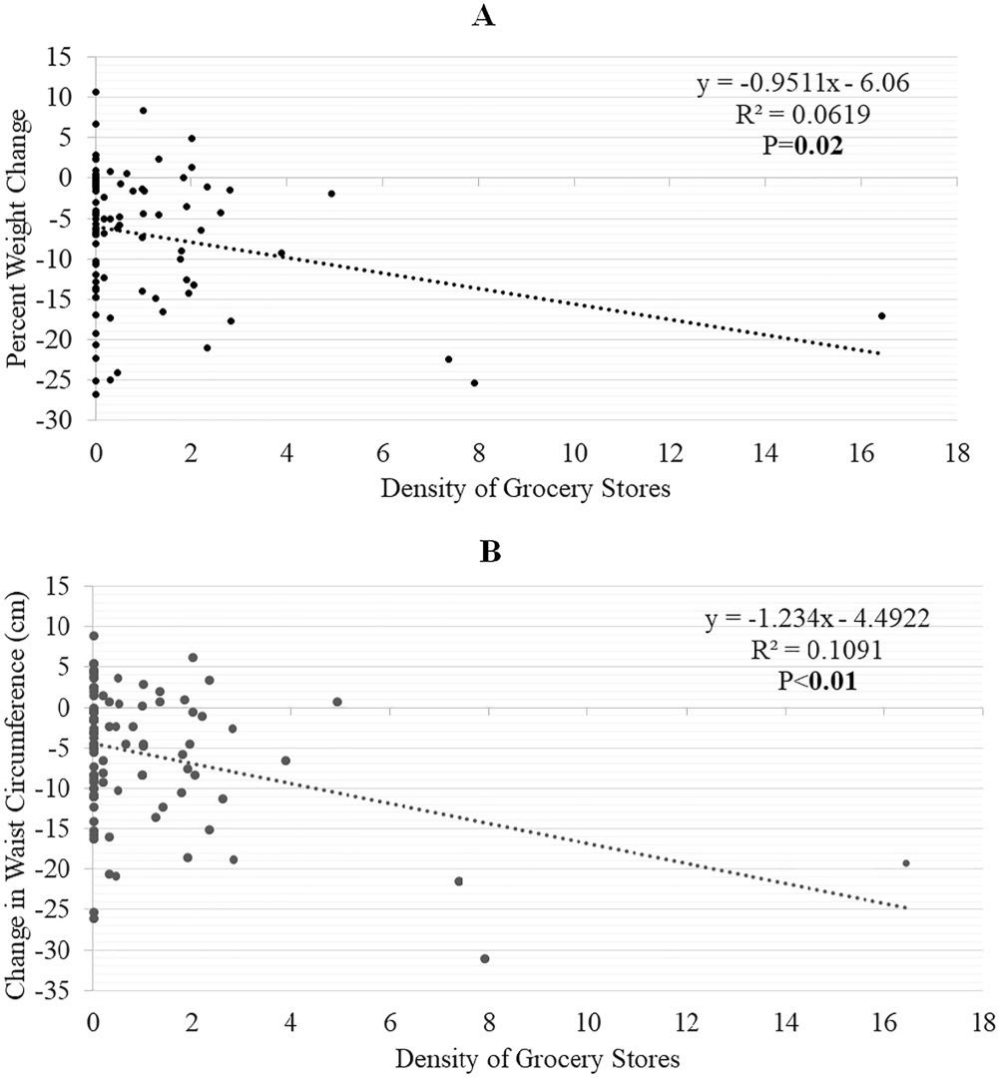
Association between density of grocery stores and changes in (A) percent weight and (B) waist circumference (WC) at 18 months. ^a^Results from simple linear regression. Bold values indicate *p*‐value < 0.05.

There was a significant association between grocery store density and changes in weight (%) and WC (Table 3): for every increase in one grocery store per square mile, % weight change decreased by 0.95 (*R* ^2^ = 0.062), and WC decreased by 1.23 cm at 18 months (*R* ^2^ = 0.109; Figure 1). No other environmental variable was associated with changes in weight (Supplementary Figure S1) or WC at 18 months (Table 3). After conducting a sensitivity analysis of the density of food outlets within each tract, plus the neighboring tracts, there was no association between community food environment variables and weight change at 18 months (Supplementary Table S1).

**TABLE 3:** Association between environment and change in anthropometric outcomes from baseline to 18 Months^a^

Environmental variable	n	Weight (%)		WC (cm)^b^	
		β (SE)	*p*‐value	β (SE)	*p*‐value
Urbanicity^c^	86				
Intercept		−7.08 (1.01)	**<0.01**	−5.83 (0.99)	**<0.01**
Suburban		1.20 (2.00)	0.55	0.87 (1.95)	0.66
City		Reference			
Walkability	93				
Intercept		−8.91 (4.00)	0.03	−6.47 (9.91)	0.10
National walkability index		0.15 (0.31)	0.62	0.06 (0.30)	0.84
Crime	93				
Intercept		−5.90 (1.49)	**<0.01**	−4.31 (1.45)	**<0.01**
High		−1.78 (2.09)	0.40	−1.96 (2.03)	0.34
Medium		−1.43 (2.12)	0.50	−2.19 (2.07)	0.29
Low		Reference			
NDI quintiles^b^ ^,^ ^c^	90				
Intercept		−5.44 (1.73)	**<0.01**	−6.23 (1.46)	**<0.01**
Below average deprivation		−1.83 (2.90)	0.53	−0.04 (2.46)	0.99
Average deprivation		−1.72 (2.72)	0.53	−0.24 (2.30)	0.92
Above average deprivation		1.26 (3.50)	0.72	3.35 (2.96)	0.26
Most deprivation		−0.91 (3.38)	0.79	0.61 (2.85)	0.83
Least deprivation		Reference			
Convenience store density	93				
Intercept		−7.41 (1.04)	**<0.01**	−6.16 (1.01)	**<0.01**
Convenience store density		0.70 (0.96)	0.46	0.78 (0.94)	0.41
Grocery store density	93				
Intercept		−6.06 (0.91)	**<0.01**	−4.49 (0.87)	**<0.01**
Grocery store density		−0.95 (0.39)	**0.02**	−1.23 (0.37)	**<0.01**
LSR density^b^	93				
Intercept		−7.00 (1.11)	**<0.01**	−5.68 (1.09)	**<0.01**
LSR density^b^		0.01 (0.26)	0.98	−0.002 (0.25)	0.99

*Note*: Bold values indicate *p*‐value < 0.05.

^a^ Results from simple linear regression.

^b^ Abbreviations are as follows: NDI: National Deprivation Index; LSR: Limited Service Restaurants; WC: Waist Circumference.

^c^ The following categories were removed from analyses due to the small sample sizes: mixed and rural categories for urbanicity; NDI not at all for NDI.

### 3.4 Association between environment and change in PA outcomes

**FIGURE 2:**
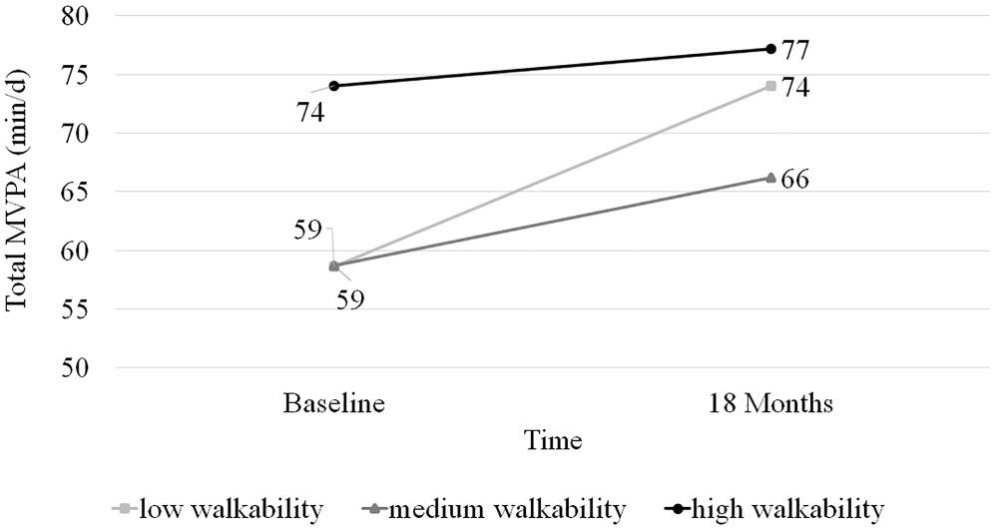
Baseline level of total moderate‐to‐vigorous physical activity (MVPA) modifies the association between walkability and changes in total MVPA at 18 months (interaction *p* = 0.03) ^a,b,^*. ^a^Results from linear regression testing an interaction between walkability and baseline level of total MVPA; low walkability: 0–12.00, medium walkability: 12.01–13.83, high walkability: >13.83; ^b^Abbreviations are as follows: MVPA: Moderate‐to‐Vigorous Physical Activity

There was a significant inverse association between walkability and changes in total MVPA at 18 months: for every 1 unit increase in walkability, change in total MVPA at 18 months decreased by 3.76 min/day (*R* ^2^ = 0.079; Table 4). Given that baseline level of total MVPA was associated with walkability (r = 0.22, *p* = 0.04), a post‐hoc analysis was conducted and found that baseline MVPA significantly moderated the association between walkability and change in MVPA. Participants living in a tract with low walkability levels and who had low MVPA levels at baseline demonstrated the greatest increase in MVPA at 18 months (Figure 2). There was a significant association between NDI and change in average total steps (count/day) at 18 months: participants in the “most deprivation” quintile significantly increased their PA by mean ± SD 2434 ± 2239 steps per day as compared to participants in the “least deprivation” quintile who only increased PA by 386 ± 2467 steps per day ((*R* ^2^ = 0.039; Supplemental Figure S2A). There were no other significant associations between other environment variables and changes in PA outcomes (Table 4). In a post‐hoc analysis, there was no correlation between walkability and average number of fitness center check‐ins per month (r = −0.04, *p* = 0.67).

**TABLE 4:** Association between built and social environment and change in physical activity outcomes from baseline to 18 Months^a^

Environmental variable	n	Total MVPA^b^		Light PA^b^		Steps		Sedentary time	
		β (SE)	*p*‐value	β (SE)	*p*‐value	β (SE)	*p*‐value	β (SE)	*p*‐value
Urbanicity^c^	74								
Intercept		5.52 (5.24)	0.30	4.71 (7.60)	0.54	670.19 (328.25)	**0.04**	−7.92 (9.37)	0.40
Suburban		−0.67 (10.34)	0.95	−5.45 (14.99)	0.72	−334.26 (647.80)	0.61	17.97 (18.48)	0.36
City		Reference							
Walkability	80								
Intercept		54.51 (18.82)	**<0.01**	8.34 (28.17)	0.77	1722.64 (1240.94)	0.17	−52.16 (35.83)	0.15
Walkability		−3.76 (1.46)	**0.01**	−0.26 (2.18)	0.91	−86.85 (96.24)	0.37	3.57 (2.78)	0.20
Crime	80								
Intercept		11.06 (7.87)	0.16	−0.56 (11.13)	0.96	475.76 (499.31)	0.34	−2.48 (14.24)	0.86
High		−5.31 (10.64)	0.62	−2.97 (15.05)	0.84	−34.26 (675.00)	0.96	9.67 (19.26)	0.62
Medium		−5.84 (10.82)	0.59	19.86 (15.30)	0.20	493.79 (686.23)	0.47	−24.68 (19.58)	0.21
Low		Reference							
NDI quintiles^b^ ^,^ ^c^	78								
Intercept		3.33 (7.21)	0.65	6.02 (10.37)	0.56	385.78 (431.16)	0.37	−4.03 (13.23)	0.76
Below average deprivation		14.90 (12.63)	0.24	−11.09 (18.18)	0.54	1008.95 (755.62)	0.19	−11.71 (23.19)	0.62
Average deprivation		−1.49 (11.86)	0.90	12.42 (17.06)	0.47	13.70 (709.23)	0.98	−12.05 (21.76)	0.58
Above average deprivation		−4.89 (14.81)	0.74	−18.36 (21.31)	0.39	−1486.63 (885.94)	0.10	28.67 (27.19)	0.30
Most deprivation		15.31 (14.81)	0.30	8.90 (21.31)	0.68	2048.27 (885.94)	**0.02**	−18.60 (27.19)	0.50
Least deprivation		Reference							

*Note*: Bold values indicate *p*‐value < 0.05.

^a^ Results from simple linear regression.

^b^ Abbreviations are as follows: MVPA: Moderate‐to‐Vigorous Physical Activity; NDI: National Deprivation Index; PA: Physical Activity.

^c^ The following categories were removed from analyses due to the small sample sizes: mixed and rural categories for urbanicity; NDI not at all for NDI.

### 3.5 Association between environment and change in dietary intake outcomes

Limited‐service restaurant density was positively associated with change in % protein intake at 18 months (*p* = 0.046; *R* ^2^ = 0.051; Figure S2B). There were no other associations between environmental variables and changes in EI or dietary macronutrient content (Table 5).

**TABLE 5:** Association between community food environment and change in energy intake outcomes from baseline to 18 Months^a^

Environmental variable	n	Energy intake (kcal/d)		Fat (%)		Carbohydrates (%)		Protein (%)	
		β (SE)	*p*‐value	β (SE)	*p*‐value	β (SE)	*p*‐value	β (SE)	*p*‐value
Crime	78								
Intercept		−283.58 (118.58)	**0.02**	−1.62 (1.86)	0.39	1.49 (2.42)	0.54	0.28 (1.22)	0.82
High		90.71 (160.04)	0.57	1.35 (2.51)	0.59	−0.20 (3.26)	0.95	0.04 (1.64)	0.98
Medium		−8.87 (161.37)	0.96	3.16 (2.53)	0.22	−4.19 (3.29)	0.21	1.29 (1.66)	0.44
Low		Reference							
NDI quintiles^b^ ^,^ ^c^	75								
Intercept		−276.85 (109.92)	**0.01**	0.05 (1.72)	0.98	−1.00 (2.14)	0.64	1.76 (1.10)	0.11
Below average		227.43 (188.11)	0.23	−0.02 (2.95)	0.99	3.04 (3.67)	0.41	−2.18 (1.88)	0.25
Average		−88.12 (173.80)	0.61	1.87 (2.72)	0.49	−4.29 (3.39)	0.21	0.66 (1.74)	0.71
Above average		−35.83 (229.92)	0.88	−0.60 (3.60)	0.87	6.45 (4.48)	0.15	−3.74 (2.30)	0.11
Most		195.21 (229.92)	0.40	−3.26 (3.60)	0.37	5.93 (4.48)	0.19	−3.21 (2.30)	0.17
Least		Reference							
Convenience stores	78								
Intercept		−241.90 (74.92)	**<0.01**	0.11 (1.18)	0.93	−0.12 (1.55)	0.94	1.03 (0.77)	0.19
Convenience store density		−22.84 (72.56)	0.75	−0.28 (1.15)	0.81	0.16 (1.50)	0.91	−0.53 (0.74)	0.45
Grocery stores	78								
Intercept		−256.59 (69.32)	**<0.01**	−0.41 (1.09)	0.71	0.47 (1.42)	0.74	0.57 (0.71)	0.43
Grocery store density		2.70 (28.21)	0.92	0.40 (0.43)	0.37	−0.54 (0.58)	0.35	0.19 (0.29)	0.51
LSR density^b^	78								
Intercept		−243.33 (81.73)	**<0.01**	−0.88 (1.28)	0.49	1.58 (1.66)	0.34	−0.28 (0.82)	0.73
LSR density		−4.08 (19.18)	0.83	0.32 (0.30)	0.29	−0.61 (0.39)	0.12	0.39 (0.19)	**0.046**

*Note*: Bold values indicate *p*‐value < 0.05.

^a^ Results from simple linear regression.

^b^ Abbreviations are as follows: LSR: Limited Service Restaurants.

^c^ The following categories were removed from analyses due to the small sample sizes: NDI not at all for NDI.

## 4 DISCUSSION

This is one of few studies to examine multiple environmental factors, recommended by the ADOPT Core Measures project,^3^ as potential environmental moderators of responses to a weight loss intervention. Similar to existing literature,^7^ findings from the present study provide mixed support for Grossman's health production theory's competing hypotheses.^6^ Results showing an association between grocery store density and greater weight loss support the complementarity hypothesis. In contrast, results indicating that participants with lower walkability increased MVPA more, and that participants living in a tract with the most deprivation demonstrated more favorable changes in average total steps provide some support for the substitution hypothesis. However, multiple, additional intervention studies are required to determine the potential importance of environmental factors in the response to weight loss interventions.

Participants living in a tract with a greater density of grocery stores lost more weight compared to participants living in a tract with a lower density of grocery stores, whereas density of convenience stores and LSRs were not associated with changes in weight or WC at 18 months. In contrast, Tarlov et al. showed some evidence that women who lived further from a supermarket lost more weight at 6 months compared to women who lived closer to a supermarket during the VA MOVE! weight management program.^16^ Among men, those who lived closer to fast‐food restaurants or convenience stores lost slightly less weight compared to those who lived further from those food outlets.^16^ But at 24 months, access to any food outlet type was not associated with weight change.^17^ Similarly, Mendez et al. found no association between density of grocery stores or restaurants and 6‐month weight change during a weight loss intervention in 127 participants.^15^ Weak or absent associations between local food environments and response to weight loss interventions may reflect widespread food availability in this population.

There was no association between the built environment (urbanicity, walkability) and changes in weight or WC. Similarly, Mench et al. found that urbanicity did not moderate weight change during a 6‐month behavioral weight loss intervention.^13^ In addition, Zenk et al. found little to no association between walkability, park access, or fitness facility access and weight change at 6, 12, 18, or 24 months among 114,256 VA MOVE! study participants.^14^ Of note, there are important differences in the PA components of these interventions, with one providing no fitness center access,^13^ another emphasizing built environment supports (e.g., use of local fitness centers and walking for transportation),^14^ and the present study providing fitness center access over 12–18 months, with little emphasis on use of built environment to facilitate weight loss efforts. However, results only represent findings from three interventions. Thus, additional interventional data are needed to elucidate whether built environment moderates weight loss response.

There was no association between the social environment (crime, NDI) and changes in weight or WC at 18 months. Similarly, Mendez et al. found no association between SES factors (poverty rate, neighborhood income) and 6‐month weight changes during a behavioral weight loss intervention.^15^ In addition, Myers et al. found that a 6‐month supervised exercise intervention improved cardiovascular health (composite score combining BMI, blood pressure, cholesterol, and glucose) for participants, regardless of their socioeconomic position (SEP; a single measure that combined self‐reported income and education). However, differences in cardiovascular health by SEP were maintained over the course of the intervention, with those who had high SEP demonstrating significantly better cardiovascular health versus those with low SEP at 6 months.^26^ Thus, interventions targeting lifestyle changes may maintain or even widen socioeconomic health disparities. Furthermore, a 10‐year longitudinal cohort study found that high levels of neighborhood crime were associated with a decrease in BMI.^12^ Importantly, the way in which SES is measured is critical, and future studies should consider this when exploring the relationship between SES and weight. For example, a review of longitudinal cohort studies found that when SES is measured with occupation, lower SES was associated with greater weight change over time, but when SES was measured using income, findings were inconsistent.^10^ The use of NDI as a measure of neighborhood SES in the present study represents a strength, as it provides a more comprehensive assessment of SES.

Baseline level of MVPA moderated the association between walkability and changes in MVPA over 18 months. Adults living in less walkable tracts had lower levels of baseline MVPA and demonstrated greater increases in MVPA at 18 months. Thus, it is possible that participants living in less walkable tracts benefited more from the PA intervention components (membership to AHWC fitness center, individualized exercise support) compared to those living in more walkable tracts. However, results from the post‐hoc analysis indicated no association between walkability and fitness center check‐ins. Similar to the present study's findings, Kerr et al. showed an inverse association between walkability and change in PA among participants in a lifestyle intervention that targeted improvements in walking and diet: men living in less walkable tracts walked less at baseline but increased their walking more compared to men living in more walkable tracts, with a similar non‐significant trend found in women.^27^ In combination, these results may support the use of interventions aiming to increase PA in participants living less walkable environments, as these populations may benefit more from the intervention. However, a limitation in both studies, the location of where PA was performed was unknown, which would be a useful addition to future research.

In terms of the association between the social environment and changes in PA outcomes at 18 months, NDI was significantly associated with changes in average daily steps. Participants in the “most deprivation” quintile demonstrated a greater increase their daily step count (from 5776 ± 1941–8210 ± 3373) as compared to participants in the “least deprivation” quintile who only increased their steps per day from 5992 ± 1867–6378 ± 1883. Thus, while participants in both groups started the intervention at a similar level of average daily steps, those living in census tracts with lower SES benefited more from the exercise intervention. These results may suggest that provision of a supervised exercise program and access to a fitness center could lessen health inequities and improve PA levels for adults living in a neighborhood with high socioeconomic deprivation. While there is a wealth of studies demonstrating a consistent, positive association between individual‐level SES (income, education, and occupation) and leisure‐time PA, the majority of existing reviews only include observational studies.^28^ Interestingly, in a recently published 10‐month diabetes prevention intervention involving diet and PA, individual‐level SES did not influence attendance, adherence, or effectiveness among 316 adults.^29^ Similarly, others have found that individual‐level SES did not influence effectiveness of diabetes prevention interventions.^30^, ^31^, ^32^ However, there is a need for additional interventional studies, among more generalizable samples (not just those with pre‐diabetes) evaluating the role of SES at the individual level and the census tract level on long‐term changes in lifestyle behaviors.

No other associations between the NDI or crime and changes in PA outcomes were observed in the present study. While several prior studies have demonstrated similar results,^33^, ^34^, ^35^ these studies all involved a PA‐specific intervention and not a comprehensive behavioral weight loss intervention. For example, Oh et al. found no association between crime (perceived and objectively‐measured) and % of prescribed walks completed among 148 Black women who participated in a walking program.^33^ Similarly, Zenk et al. found no association between violent crime (measured objectively) and % of prescribed walks completed among 252 Black women who participated in a walking intervention.^34^ In contrast, Sallis et al. found an inverse association between neighborhood crime (perceived) and change in PA minutes per week among 861 participants in an activity counseling trial.^35^ The major difference between the present study and the one by Sallis et al. is that measure of crime used in the present study is objective versus perceived. Including both objective and perceived measures of crime and safety will be important to elucidate the role of social environment on changes in PA in future interventions.

Adults living in a tract with the highest level of deprivation demonstrated a reduction in % protein intake at 18 months as compared to adults living in a tract with the least deprivation. These results may suggest that adults living in tracts with the most deprivation showed modest improvements in macronutrient intake that were more in‐line with the intervention recommendations (∼25% fat, ∼52.5% carbohydrates, and ∼22.5% protein), though this did not translate to significant reductions in EI. In two cross‐sectional studies, lower SES (occupation, housing, and education) was significantly associated with lower protein intake.^36^, ^37^ The authors are unaware of any other studies that have investigated the association between NDI and changes in dietary intake outcomes in the context of a behavioral weight loss intervention.

Adults living in a tract with a higher density of LSRs were more likely to increase their % protein intake at 18 months compared to adults living in tracts with a lower LSR density. In contrast to the present study's findings, Barnes et al. found no association between frequency of fast‐food consumption and % protein intake, both cross‐sectionally and prospectively over 6 months in a free‐living sample.^38^ There was no association between convenience store density or grocery store density and changes in EI or dietary macronutrient intake. Alternatively, Wedick et al. found that proximity to health food stores was associated with greater improvements in consumption of fiber and fruit and vegetables at 1 year among 204 adults with obesity and metabolic syndrome who received a dietary intervention.^39^ Similarly, Gustafson et al. found that participants randomized to receive a 16‐week dietary intervention and who lived in neighborhoods with no supermarkets had a greater increase in fruit and vegetable intake as compared to controls without a supermarket in their neighborhood.^40^ One reason for these differences may be that only changes in total EI and macronutrient intake were assessed and not changes in specific food types.

Limitations of this study include the small sample size, modest variation in urbanicity, the relatively homogenous sample (82% female, 75% white), and reliance on self‐reported dietary intake.^41^ Participants were excluded from the study if they lived or worked >20 miles from the university. Thus, rural areas were not well represented. The impact of the community food environment was examined based on tract‐level density (counts/mi^2^); however, a recent publication argues that future studies should use tailored buffer‐based measures based on community type in order to define food environment.^42^ Thus, estimates may be smaller than if tailored buffers had been used. Furthermore, predefined areal units (i.e., census tracts) were used to define environmental exposure. This issue, defined as the “uncertain geographic context problem” by Mei‐Po Kwan, suggests that findings about the effects of area‐based attributes on individual behaviors or health outcomes could be misrepresented because a census tract does not capture the true boundary in which adults shop for food.^43^ In addition, use of such administrative units does not capture other important dynamic, contextual factors that influence food intake (e.g., time constraints, temporal availability of food outlets, social contexts).^43^ Lastly, while there is little existing evidence of the predictive value of the NIH ADOPT environmental measures for obesity treatment outcomes, the implementation of these environmental factors among weight loss studies represents an initial step in building systematic evidence on the role of environment for obesity outcomes.^3^ Future studies should explore the predictive value of the NIH ADOPT environmental factors and consider a more complete analysis of how participants interact with their environments when examining the relationship between food environment and health outcomes.

Results indicate that environmental factors may account for some of the variability (<11%) in response to behavioral weight loss interventions. It will be important to continue to assess the impact of environmental factors on weight loss outcomes in additional, interventional studies with assessments that capture the dynamic, contextual environmental factors that influence EI and PA behavior. Pooling data across multiple weight loss interventions will improve knowledge regarding whether environment moderates weight loss outcomes. This line of research could lead to policies that support environmental changes to enhance environmental supports (e.g., improving access to grocery stores) and/or the development of more tailored weight loss interventions that account for environmental factors (e.g., providing additional PA support for individuals who live in neighborhoods with low walkability and/or a greater emphasis on improving knowledge of local environmental supports within the behavioral intervention content) to improve long‐term obesity treatment outcomes.

## AUTHOR CONTRIBUTIONS

VAC conceived of and designed the parent trial, and obtained funding for the parent trial that supplied data used in this secondary data analysis. VAC wrote the protocol and acquired the data. BS, DGS, and DB provided the environmental variables (urbanicity, walkability, crime, NDI, and density of grocery stores, convenience stores, and LSRs) for the state of Colorado. ST and DMO performed the statistical analyses. All authors assisted with interpretation of the data. ST, DMO, and VAC drafted the manuscript. ST and DMO generated tables and figures. All authors were involved in writing and revising the manuscript and approved the final version of the manuscript.

## CONFLICT OF INTEREST

The authors declare no conflicts of interest.

## CLINICAL TRIAL REGISTRATION

NCT01985568.

## DISCLOSURE

Nothing to disclose.

## Supporting information

Supplementary Material
