# Supplementary material for: Impact of the built, social, and food environment on long‐term weight loss within a behavioral weight loss intervention
Source: Obes Sci Pract. 2022 Nov 3;9(3):261–73. doi: 10.1002/osp4.645 (PMC10242259; doi:10.1002/osp4.645)
Supplement: Supplementary file 1 — Supplementary Material [file OSP4-9-261-s001.docx]

**Supplemental Information**

**TITLE:** Impact of the Built, Social, and Food Environment on Long-term Weight Loss within a Behavioral Weight Loss Intervention

**AUTHORS:** Selam Tewahade ^1^**,** David Berrigan ^2^, Beth Slotman ^3^, David G. Stinchcomb ^3^, R Drew Sayer ^4^, Victoria A. Catenacci ^5, 6^, and Danielle M. Ostendorf ^5, 6^

**AFFILIATIONS:**

^1^ Department of Epidemiology, Colorado School of Public Health, University of Colorado Anschutz Medical Campus, Aurora CO, USA

^2^ Division of Cancer Control and Population Sciences, National Cancer Institute, Bethesda, MD, USA

^3^ Westat, Rockville MD, USA

^4^ Department of Nutrition Sciences, University of Alabama at Birmingham, Birmingham, AL USA

^5^ Division of Endocrinology, Metabolism, and Diabetes, Department of Medicine, University of Colorado Anschutz Medical Campus, Aurora CO, USA

^6^ Anschutz Health and Wellness Center, Department of Medicine, University of Colorado Anschutz Medical Campus, Aurora CO, USA

**CONTACT INFORMATION:**

Danielle M. Ostendorf, PhD

Anschutz Health and Wellness Center

University of Colorado Anschutz Medical Campus

123458 E. Montview Blvd. Mailstop C263

Aurora, CO 80045

[Danielle.ostendorf@cuanschutz.edu](mailto:Danielle.ostendorf@cuanschutz.edu)

**Supplementary Table S1.** Sensitivity Analysis of Association between Food Environment (Tract + Neighbors) and Change in Weight (%) from Baseline to 18 Months ^a^

| Environmental Variable | n | Weight (%) | |
| --- | --- | --- | --- |
|  |  | Beta Estimate (SE) | *P*-value |
| **Convenience Store Density** | 93 |  |  |
| Intercept |  | -6.93 (1.43) | **<0.01** |
| Convenience Store Density |  | -0.11 (2.66) | 0.97 |
| **Grocery Store Density** | 93 |  |  |
| Intercept |  | -7.34 (1.15) | **<0.01** |
| Grocery Store Density |  | -0.54 (1.14) | 0.64 |
| **LSR Density** ^b^ | 93 |  |  |
| Intercept |  | -6.77 (1.35) | **<0.01** |
| LSR Density ^b^ |  | -0.09 (0.47) | 0.84 |

**Legend for Supplementary Table S1:**

^a^ Results from simple linear regression.

^b^ Abbreviations are as follows: LSR: Limited Service Restaurants.


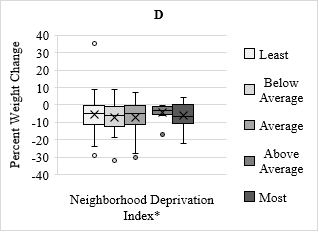

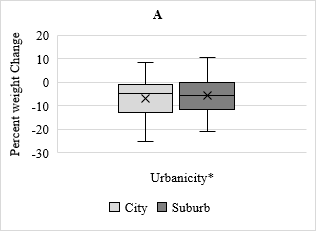

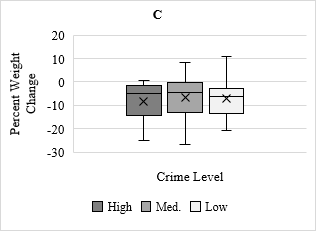
**Supplementary Figure S1.** Association between percent weight change and (A) urbanicity, (B) walkability, (C) crime, (D) Neighborhood Deprivation Index, (E) convenience store density, and (F) LSR density.

*P*=0.90

*P*=0.55

*P*=0.67

**Legend for Figure S1:**

^a^ Results from simple linear regression. Abbreviations are as follows: NDI: National Deprivation Index; LSR: Limited Service Restaurants. In the box and whiskers plot in figures A, C, and D, the horizontal line represents the median score, and the X represents the mean score. ^*^The following categories were removed from analyses due to the small sample sizes: mixed and rural categories for urbanicity; NDI not at all for NDI.


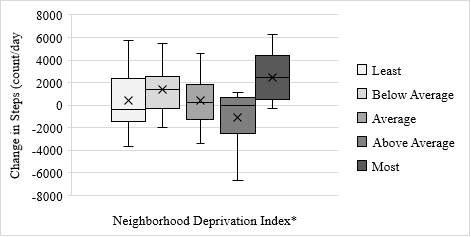
**Supplementary Figure S2:** Association between (A) NDI and change in steps, and (B) density of LSRs and change in protein intake at 18 months ^a-b^

*P*=**0.02**

**A**

**B**

**Legend for Figure S2:**

^a^ Results from simple linear regression. In the box and whiskers plot in figures A, and B, the horizontal line represents the median score, and the X represents the mean score. ^*^The following categories were removed from analyses due to the small sample sizes: NDI not at all for NDI.

^b^ Abbreviations are as follows: NDI: Neighborhood Deprivation Index; LSR: Limited Service Restaurants.
